# Hip Fracture Nonunions: Diagnosis, Treatment, and Special Considerations in Elderly Patients

**DOI:** 10.1155/2018/1912762

**Published:** 2018-11-25

**Authors:** Sharon Babcock, James F. Kellam

**Affiliations:** ^1^Department of Orthopaedic Surgery, Wake Forest School of Medicine, Winston Salem, NC, USA; ^2^Department of Orthopaedic Surgery, UT Health Science Center at Houston, Houston, TX, USA

## Abstract

In the United States, more than 300,000 hip fractures occur annually in the elderly population with associated significant morbidity and mortality. Both intracapsular and extracapsular hip fractures have inherent treatment challenges and therefore are at risk of nonunion complications. A systematic assessment including radiographic, metabolic, and infectious evaluations should be completed for all patients suspected of nonunion. Failed internal fixation of intracapsular hip fractures is typically treated with arthroplasty, while extracapsular proximal femur nonunions may be amenable to revision internal fixation or arthroplasty. While not a classic hip fracture, bisphosphate associated subtrochanteric femur fractures affect a similar patient population and are historically difficult to treat. Atypical subtrochanteric femur fractures are at increased risk of nonunion given the altered biologic environment secondary to bisphosphonate use; therefore adjuvant therapies may be beneficial in setting of revision fixation. Having a thorough understanding of nonunion risks, recognition, evaluation, and treatment is necessary for appropriate patient care.

## 1. Introduction

Hip fractures in the elderly occur in significant numbers, with 1.6 million fractures worldwide annually and a projected increase to over 6 million hip fractures yearly by 2050 [1, 2]. Hip fractures can be broken down into two generic categories: intracapsular and extracapsular, depending on where the fracture occurs about the proximal femur. Femoral neck fractures are classified as intracapsular, while extracapsular hip fractures can be further broken down into intertrochanteric and peritrochanteric, with possible subtrochanteric extension depending on the fracture line exit point about the greater and lesser trochanters. An accepted terminology to encompass all extracapsular hip fractures is peritrochanteric femur fracture, with the fracture occurring about the greater and lesser trochanter and not extending greater than 5cm from the lesser trochanter. Pure subtrochanteric femur fractures do not involve the area about the trochanters, instead involving the proximal third femoral shaft. This fracture type is fraught with its own reduction and treatment challenges and is not included within the group of fractures classically considered “hip fractures.” In contrast, atypical femur fractures (AFFs), otherwise known as subtrochanteric femur fractures associated with prolonged bisphosphonate, occur in a similar patient population as hip fractures and therefore were included for this review.

In a study reviewing the United States 2008 Nationwide Emergency Department Sample emergency room visits, it was found that more than 90% of 341,000 hip fractures occurred in patients over the age of 60 and that overall the rate of peritrochanteric femur fractures compared to femoral neck fractures was 2:1 [3]. It is well accepted that hip fractures, whether peritrochanteric or femoral neck in morphology, generally necessitate surgical treatment if the patient is medically stable to survive the operation and will benefit from pain relief and improved mobility provided by the surgical intervention. Typically peritrochanteric femur fractures are treated with internal fixation, while femoral neck fractures are treated with either internal fixation or arthroplasty, but controversy exists regarding which treatment is better [4, 5]. The morbidity and mortality, along with the socioeconomic impact of hip fractures, are substantial, and complications of fracture nonunion and fixation failure only compound these effects.

All hip fractures treated operatively with internal fixation or treated nonoperatively have the potential to go onto nonunion. Those fractures treated with a type of arthroplasty are not at risk for nonunion, as the proximal portion of the fractured femur is discarded to allow for implantation of the arthroplasty replacement. The US Food and Drug Administration (FDA) defines a nonunion as a fracture 9 months from injury that shows no visible progressive healing for 3 months, yet this is not always a practical definition. Therefore, additional accepted definitions for nonunion are the following: a fracture that in the opinion of treating physician has no possibility of healing without further intervention [6] or a fracture that has shown no radiographic progression over 3 consecutive images in a patient who has clinical symptoms consistent with nonunion. The causes of nonunion are commonly multifactorial, consisting of biological, mechanical, injury, and patient factors that all contribute to putting a fracture at risk of nonunion [7]. Basic biological requirements for fracture healing are a combination of mechanical stability from appropriate fixation, adequate bony vascularity, and osteoprogenitor and growth factor cells, as well as bone-to-bone contact of the fracture fragments [8]. When a combination of these requirements is not met, nonunion may occur. Classically, hypertrophic nonunions occur when there is adequate vascularity but inadequate fracture stability, and this results in abundant nonbridging callus formation at the fracture site. Atrophic nonunions lack adequate blood supply at the fracture site, which may have been injured at the time of trauma or have been iatrogenic secondary to poor soft tissue handling at the time of surgery. These nonunions show no callus at the fracture site and evidence of bone resorption. Oligotrophic nonunions, also known as the “surgeon's nonunion,” are characterized by a diastasis at the fracture site, minimal callus, and variable vascularity and mechanical stability [6].

Femur nonunions result in decreased mental and physical health, along with debilitating impacts on activities of daily living [9]; therefore when treating hip fracture patients, it is imperative to monitor for adequate healing postoperatively and begin an appropriate nonunion evaluation for those patients who meet the previously quoted definitions. Failure to recognize a nonunion could result in the catastrophic complication of fixation failure and need for revision surgery in an emergency setting [10], compared to intervention or revision surgery on a planned timeline if the problem is recognized earlier.

## 2. Nonunion Evaluation

Hip fracture nonunion should be suspected in patients who continue to have groin or thigh pain with ambulation, sitting, or transfers that cannot be explained by other etiologies. Radiographs should be closely evaluated for signs of hardware loosening with presence of a surrounding “halo” about implants, as well as fracture shortening, or lack of expected callus formation or bridging of fracture fragments [10, 11]. Radiographs of the entire limb should be obtained to evaluate overall limb alignment. Computed tomography (CT) scan is typically indicated to better characterize the nonunion, estimate the cross sectional area of bridging callus, and evaluate for rotational deformities [6]. Infection must be ruled out, as indolent infection can contribute to fracture nonunion. White blood cell (WBC) count, C-reactive protein (CRP), and erythrocyte sedimentation rate (ESR) should be obtained, and their elevation, especially when combined, is a strong predictor of infection [7]. Additionally, intraoperative cultures should be obtained at the time of revision surgery even if there are no clinical signs of infection, as 20% of patients in a large multicenter series were shown to have positive cultures, with only 9% felt to be contaminants [12]. If infection is confirmed in the initial laboratory evaluation and surgical intervention is deemed necessary and safe, multiple treatment options exist. Removal of hardware and culture-specific antibiotics should be considered in all operative candidates. Resection arthroplasty, staged debridement with revision internal fixation, staged debridement with arthroplasty, and single stage revision arthroplasty are all viable options. While staged debridement with antibiotic spacer placement followed by arthroplasty after eradication of infection is a reliable treatment option [13, 14], it is important to be aware of the risks associated with revision arthroplasty surgery. Ford et al. evaluated 80 patients with an average age of 64 who began the process for two-stage exchange arthroplasty in setting of infection. 30% had a serious complication, including 3 perioperative deaths, 30% required an additional operation, 18% never underwent reimplantation, and 27% became reinfected [15].

In addition to ruling out infection, a metabolic and endocrine assessment should be completed in the setting of nonunion. Abnormalities within vitamin D and calcium processing pathways, thyroid function, and other hormonal interactions have all been associated with delayed fracture healing and may be present in those patients presenting with nonunion [16, 17]. We recommend an initial evaluation including the laboratory tests listed in Table 1. Based on the results of these screening tests, if a more specific evaluation is required, we recommend referral to an endocrinologist. There, further testing can be performed to evaluate levels of estrogen, testosterone, cortisol, and additional specific labs depending on the associated patient complaints and symptoms [7, 10].

Adequate treatment of any metabolic abnormalities recognized during this evaluation is recommended, with some nonunions healing without any additional surgical intervention following the metabolic correction [16]. For example, it was shown that hypoalbuminemia and lymphocytopenia are both independent predictors of fixation failure in nondisplaced femoral neck fractures [18]; therefore correction of such laboratory values may theoretically decrease failure and therefore reoperation and nonunion rates. Even for those patients without vitamin D deficiency, vitamin D supplementation has been shown to decrease the risk of hip fractures and increase proximal femur bone density [19], and a recent study has shown the cost effectiveness in calcium and vitamin D supplementation in fracture patients while attempting to reduce the nonunion rate [20]. Therefore, while completing a thorough metabolic workup in the setting of nonunion is crucial, correcting any preexisting abnormalities and deficiencies at the time of the index fracture may have an overall positive effect on fracture healing.

## 3. Femoral Neck Fracture Nonunion

The optimal treatment for elderly intracapsular femoral neck fractures continues to remain controversial. It is generally taught that nondisplaced femoral neck fractures (Garden types I and II) undergo internal fixation with a cannulated screw construct, while displaced femoral neck fractures (Garden types III and IV) are treated with arthroplasty [21–23]. While internal fixation affords the advantage of maintaining the patient's own femoral head, less surgical trauma, and, in very frail patients, a slight decrease in overall morbidity and mortality [24], there are high rates of complications for those undergoing fixation, including avascular necrosis, delayed union, nonunion, fracture shortening, and increased rate of reoperation [4, 25]. A recent meta-analysis of 34 articles comparing THA, hemiarthroplasty, and osteosynthesis for displaced femoral neck fractures in patients over the age of 60 showed high revision rates for fractures treated with internal fixation compared to both THA and hemiarthroplasty [5]. This echoed a previous meta-analysis of 106 studies, which showed a reoperation rate of 20-36% within two years [25]. The international, multicenter, randomized FAITH trial showed that, for all types, low energy femoral neck fractures in patients over 50 years of age, whether treated with sliding hip screw or cannulated screws, there was a 21% reoperation rate, with an average of 15% undergoing revision to either total hip arthroplasty or hemiarthroplasty. Only 67% of all fractures healed by 24 months, and, of those that healed, nearly 30% had fracture shortening of more than 5mm [26]. A subsequent study determined that female sex, higher body mass index (BMI), displaced fracture, prominence of screws at the lateral femoral cortex, screw penetration, high placement of lag screw on immediate postoperative images, and smokers treated with cannulated screws were at increased rate of revision surgery [27]. Similarly, Yang et al. found a significantly increased odds ratio of 2.93 for nonunion in displaced femoral neck fractures compared to nondisplaced fractures in a study of 202 patients with an average age of 64.5. Additional risk factors for nonunion were less than anatomic reduction and traditional triangle compared to inverted triangle configuration of cannulated screws [28]. A decrease in contralateral hip bone mineral density (BMD) has also been shown to increase the risk of nonunion in displaced femoral neck fractures treated with cannulated screws [29]. It is important to remember these risk factors for complications and nonunion in elderly femoral neck fractures treated with internal fixation and to pursue appropriate interventions if necessary.

**Figure 1:**
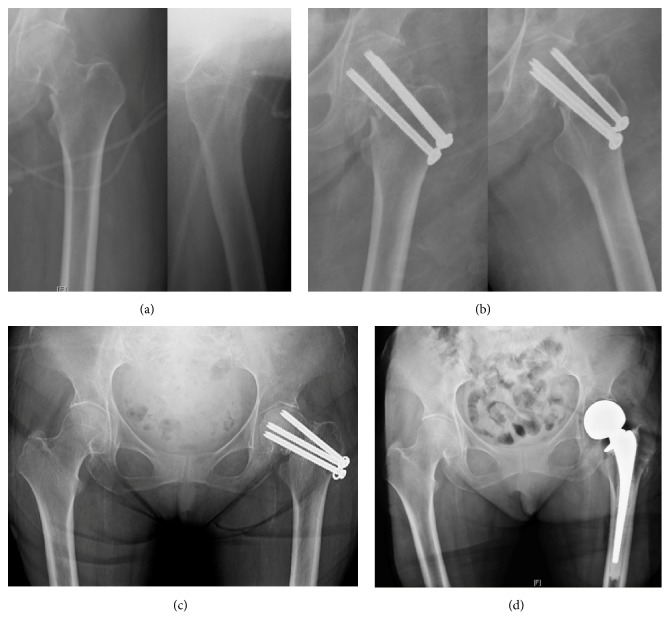
(a) A 61-year-old female sustained a syncopal fall at home resulting in a displaced left transcervical femoral neck fracture. She has a history of multiple medical comorbidities including end-stage renal disease on hemodialysis, diabetes mellitus, and hypertension. She subsequently went into PEA arrest twice during transport to the hospital. She was intubated and sedated on arrival. She remained critically injured in the Intensive Care Unit and was deemed an extremely high risk for surgery; therefore surgical intervention was delayed until the patient was medically stable. On hospital day 8, the patient was cleared by the medical service for surgical intervention of her displaced femoral neck fracture, and after extensive conversation with the patient and her family, consent was obtained and surgery was pursued with the plan for hemiarthroplasty given her fracture displacement and delayed presentation to the OR. After induction of anesthesia, the patient was deemed too instable to undergo a hemiarthroplasty, so conversion to closed reduction with percutaneous cannulated screws was chosen, aware of the chance of failure, but choosing this given her medical instability and high risk (b). She was followed in the clinic and over the following 7 months continued to have groin pain with ambulation and radiographic signs of hardware failure and nonunion (c). During this time, she had extensive medical optimization in anticipation of revision surgery. When deemed medically optimized and cleared for surgery, after discussion with the patient and her family, she was brought back to the OR for hardware removal and left hip hemiarthroplasty (d). She tolerated the procedure without complications and is ambulating with minimal pain postoperatively.

Usually femoral neck nonunions in physiologically young patients are treated with methods to salvage and maintain the patient's own native femoral head, and typically this is accomplished with a valgus intertrochanteric osteotomy. This procedure improves the mechanical environment of the nonunion, creating compressive rather than shear forces across the nonunion [11, 23, 30, 31]. In elderly patients with femoral neck nonunions, the accepted revision treatment method is arthroplasty given the reliability of outcomes, potentially decreased surgical insult, and ability for immediate weight bearing (Figure 1) [4, 11, 23, 32]. There is debate regarding the decision between total hip arthroplasty (THA) and hemiarthroplasty, but if the articular cartilage of the femoral head or acetabulum has been damaged, total hip arthroplasty is recommended [11]. Mabry et al. evaluated 84 patients who underwent Charnley cemented THA for nonunion from 1970 to 1997 with an average follow-up of 12 years. There was a 93% survival free revision rate at 10 years and 76% at 20 years, with 96% of the 72 who never underwent revision having no or mild hip pain, indicating THA as a reliable long-term treatment option for femoral neck nonunions [32]. There is documented increased risk of dislocation following THA for failed osteosynthesis of femoral neck fractures compared to primary THA [32, 33]; therefore it is recommended that the patient undergo conversion arthroplasty surgery by a surgeon who is trained in revision arthroplasty.

## 4. Peritrochanteric Femur Fracture Nonunion

Peritrochanteric femur fractures typically occur in a physiologically older population than femoral neck fractures, account for about 50% of hip fractures, and include all proximal femur fractures that occur from the extracapsular femoral neck area to below the lesser trochanter within 5cm [34]. Stable 2-part intertrochanteric femur fractures with an intact lateral cortex and typical fracture obliquity were classically treated with a sliding hip screw (SHS). Instable 3- or 4-part peritrochanteric femur fractures or those with a reverse obliquity fracture pattern (fracture from the proximal medial cortex exiting distally on the lateral femoral cortex) are treated with cephalomedullary nail (CMN). Overall, there has been an increased use of CMNs for all peritrochanteric femur fractures, independent of the perceived stability [35, 36]. The results of a recent randomized controlled trial confirmed this treatment algorithm, showing greater healing complication, revision procedures, femoral shaft medicalization, and associated pain for multifragmentary intertrochanteric femur fractures treated with SHS compared to intramedullary nail [37]. Similarly, Chehade et al. saw an increased rate of reoperation, as well as increased mortality, in unstable peritrochanteric femur fractures, especially those treated with the Australian Austofix intramedullary nail secondary to Z-effect phenomenon [38]. Baldwin et al. summarized current treatment controversies for cephalomedullary nailing of intertrochanteric femur fractures, stating that there is no difference in fixation failure or need for revision surgery when comparing short versus long CMNs, helical blade versus lag screw, or locked versus unlocked nail in the setting of a stable fracture pattern. Tip apex distance <25mm continues to be the most important factor to decrease implant cutout, regardless of blade or screw type. Unstable peritrochanteric femur fractures are recommended to be treated with distal interlocking screws for improved biomechanical properties, including torsional resistance [35]. Fracture reduction continues to be imperative for overall outcome, as fractures with “poor” reduction (varus malalignment on AP image, >20 degrees angulation on lateral image, or >4mm displacement) had significantly shorter time to failure than those with “adequate” or “good” reduction [39]. While choosing the correct implant specific to fracture morphology and obtaining a good reduction improve likelihood of fracture union, nonunions of peritrochanteric hip fractures unfortunately occur and various treatment options exist.

If nonunion or implant failure results following internal fixation of a peritrochanteric femur fracture, there are a number of factors to consider, which will assist in determining the appropriate revision treatment for the patient. First, patient's functional level, goals of care, and life expectancy must be evaluated. If revision surgery is desired, then, as with all nonunions, metabolic and infectious evaluations are begun. Next, evaluating proximal femoral bone stock and integrity of the articular cartilage will help determine if revision internal fixation is possible or if conversion to arthroplasty is the appropriate treatment.

**Figure 2:**
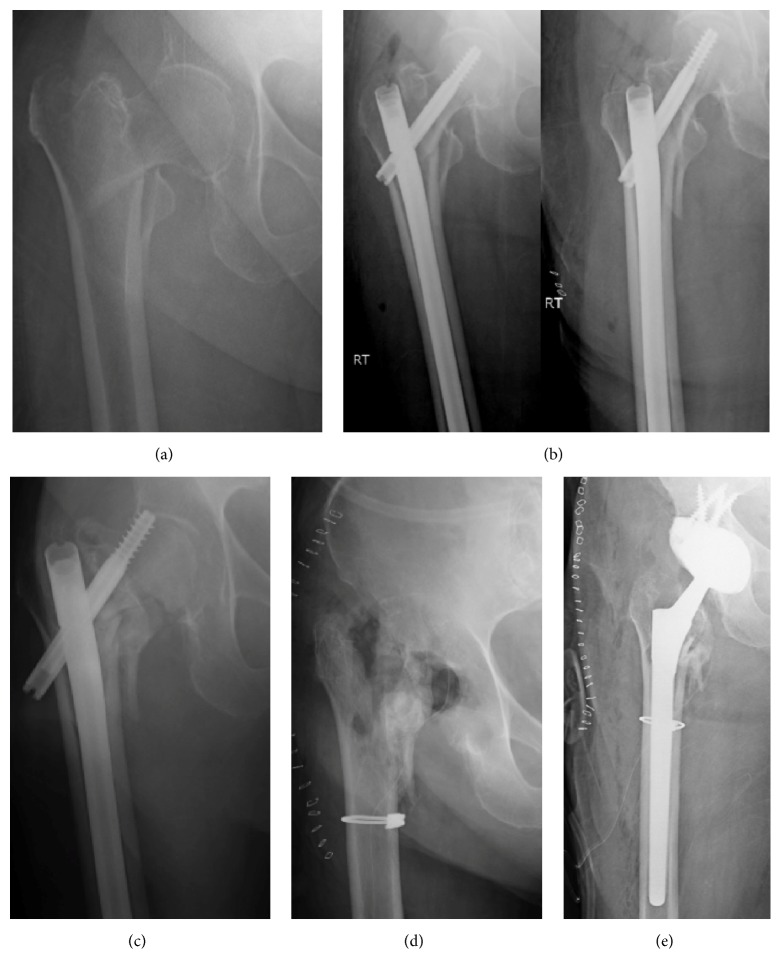
(a) A 85-year-old active female sustained a fall from standing height onto her right hip resulting in an unstable displaced intertrochanteric femur fracture. (b) She underwent locked cephalomedullary nailing within 24 hours of the injury at an outside institution. The neck-shaft angle measures 119 degrees with a 130-degree implant. Note the cranial endpoint for the compression screw. (c) She was referred to an arthroplasty specialist 7 months later with catastrophic failure, screw cutout, and associated acetabular defect. (d) After a long discussion with the patient and her desire for pain relief and ability to ambulate, the decision was made for removal of hardware with femoral head resection and obtainment of cultures. (e) Two days later, cultures were negative for infection, and she was brought back to the operating room for total hip arthroplasty implantation with acetabular augment to correct defect created by the compression screw cutout. A long diaphyseal-fitting stem was used given the poor proximal bone stock, and a cable was placed to support the lateral cortex from lag screw stress riser. The patient is ambulating without pain or assist device 10 months postoperatively.

Screw cutout is the common mode of failure, commonly resulting in cartilage damage and necessitating conversion to THA. Additionally, if there is evidence of hip arthritis, THA is advocated [10, 11]. Depending on the amount of proximal femur bone stock remaining and prior stress risers along the shaft created by the previous fixation implant, a calcar-replacing implant, long stem implant, or a megaprosthesis (proximal femur replacement) may be required (Figure 2) [11, 39–41]. The majority of patients treated with calcar-replacing prostheses for intertrochanteric nonunion had significant reduction in pain and the returned ability to ambulate [40, 41]. If adequate proximal femoral bone stock is present and hip joint integrity is maintained, revision operative fixation should be considered. Correction of fracture malreduction, improving fracture stability, and increasing biology to the fracture may all be necessary depending on the contributing factors to nonunion. Thorough debridement of the nonunion site should be completed prior to revision fixation, with the option for bone grafting at the nonunion site if deemed necessary [6, 10]. In a study of 1360 peritrochanteric femur fractures treated with CMN, 20 patients underwent revision for nail fatigue failure. 40% were treated with revision CMN, 30% were treated with arthroplasty or megaprosthesis, and the remaining underwent revision fixation with a proximal femur locking plate. Overall, there was a higher rate of rerevision in the proximal femur locking plate group and a longer time to radiographic union. Regardless of revision treatment modality, 1-year mortality rate following revision fixation was 30% [39]. Ultimately, one must take the entire patient into account when choosing the appropriate intervention for peritrochanteric femoral nonunion, as the mortality rate is similar to that of the index hip fracture, and postoperative rehabilitation may vary depending on treatment modality and weight bearing restrictions.

## 5. Atypical Subtrochanteric Femur Fracture Nonunion

Although not classically included in the encompassing category of “hip fracture,” atypical subtrochanteric femur fractures related to long-term bisphosphonate use affect a similar patient population and can be fraught with the same healing challenges of femoral neck and peritrochanteric femur fractures. Atypical femur fractures (AFFs) are located between the lesser trochanter and the supracondylar flare of the femur, although they are most commonly located in the subtrochanteric region. They must have 4 of 5 major criteria as defined by the American Society for Bone and Mineral Research (ASBMR): (1) pathologic or low energy injury, (2) fracture line starting at lateral cortex and being transverse or short oblique in orientation, (3) no or minimal comminution, (4) complete fractures extending through both cortices and may create a medial spike, while incomplete fractures involve only the lateral cortex, and (5) lateral cortical beaking being present and indicative of local periosteal thickening [42]. Al-Ashqar et al. summarize the underlying pathogenesis of AFFs secondary to prolonged bisphosphonate use causing reduced osteoblast and osteoclast activity resulting in “severely suppressed bone turnover,” accumulation of microdamage without the normal reparative process, and unregulated mineralization, leading to increased brittleness [43]. Odvina et al. first described severely suppressed bone turnover in 2005, noted in patients on long-term alendronate therapy with spontaneous nonspinal fractures, whose cancellous bone showed low osteoblastic and osteoclastic activity [44]. Therefore bisphosphonate usage should cease immediately at the time of fracture.

**Figure 3:**
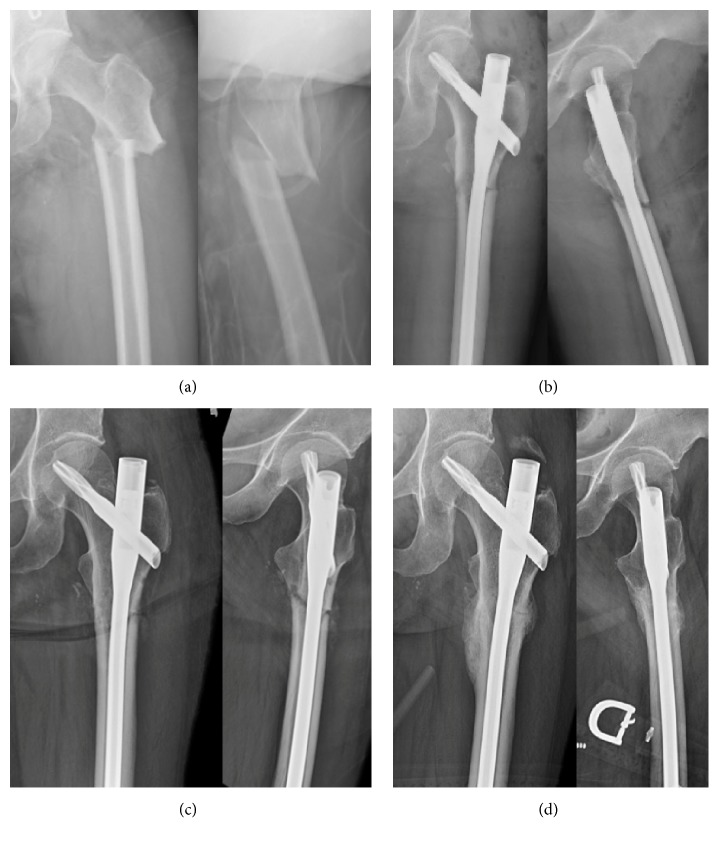
(a) Atypical left femur fracture sustained after a fall from standing in a 58-year-old female with history of bisphosphonate use. (b) She underwent closed reduction and cephalomedullary nailing with a slightly medial start point to induce slight valgus alignment with the trochanteric proximal nail bend. (c) At 6 weeks postoperatively, she denies pain and is ambulating without assistance. Fracture alignment is maintained. (d) At 6 months, she has radiographic union and is back to all activities.

The risk of fracture is associated with increased duration of bisphosphonate use, and Asian ethnicities are at increased risk secondary to increased femoral bowing and increased lateral cortical stresses [43]. Because of the altered bone physiology, multiple studies have shown delayed healing, with increased nonunion rates of around 30% [44–47], so performing a well-done index procedure is critical. Cephalomedullary nailing has been shown to have better outcomes than plate fixation, with plate failure averaging 30% [46, 48]. Reduction continues to be critical to outcomes (Figure 3). For subtrochanteric femur fractures, neutralizing the typical deformity of flexion, abduction, and external rotation of the proximal fragment is necessary, and AFF transverse or short oblique morphology results in an inherently unstable pattern with decreased bony contact. Cho et al. evaluated 48 fractures in 42 patients and found an overall primary healing rate of 68.7%, with 15 cases of failure. Fractures fixed in 5 degrees or more of malreduction in any plane had a significantly higher likelihood of healing failure, with a neck-shaft angle of at least 125.6 degrees, 4.4 degrees less than varus angulation compared to the uninjured contralateral hip, and 5.5 degrees less than sagittal angulation [45].

**Figure 4:**
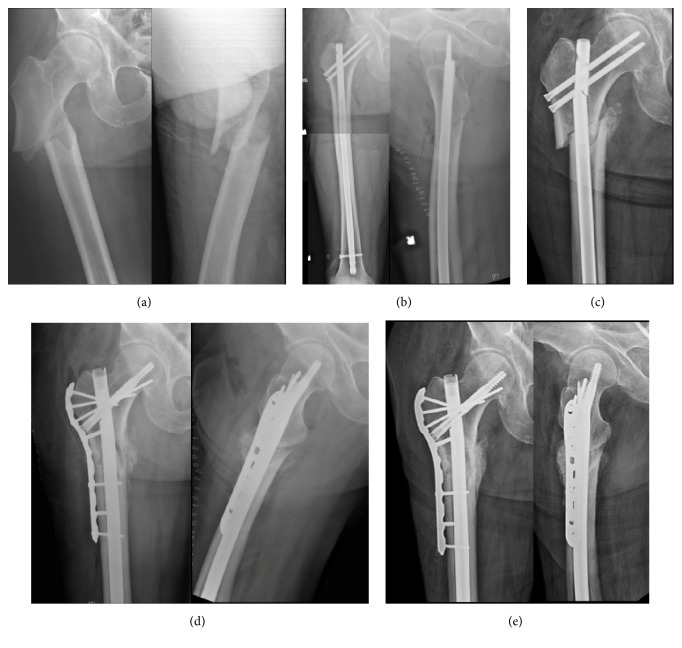
(a) Atypical right subtrochanteric femur fracture created in a 68-year-old male with 8-year history of bisphosphonate use. (b) He underwent open reduction internal fixation with a locked piriformis reconstruction nail within 24 hours of injury. Note anatomic alignment immediately postoperatively. (c) At 8 weeks postoperatively, he had increased pain in his hip and felt a pop. New radiographs reveal hardware failure with nail fracture and varus collapse. (d) Given his adequate proximal femoral bone stock, he underwent revision open reduction internal fixation with removal of hardware, locked cephalomedullary nailing, and augmentative plating with autologous bone grafting of the nonunion site. (e) At 6 months postoperatively, he has radiographic signs of union and is pain-free and returned to all previous activities.

Average time to union for atypical subtrochanteric femur fracture ranges from 7.3 to 10.7 months [45, 47, 48], and intervention in the setting of delayed union prior to implant failure should be considered. In the setting of atrophic nonunion without malreduction, autologous bone grafting has been successful in obtaining union without hardware revision [45]. Correcting malreduction, even inducing a slight amount of valgus, will improve mechanical properties across the fracture site to aid in healing (Figure 4). Improving the biologic environment is also possible with teriparatide, an anabolic osteoporosis medication that stimulates bone formation and promotes growth factor production for fracture healing. A meta-analysis of 251 patients with osteoporosis treated with teriparatide suggested faster fracture healing times and improved functional outcomes [49], with the possibility that starting treatment immediately at the time of fracture is more effective than waiting 6 months [50]. Similar to adding biology with autologous bone graft in delayed union, a case report illustrates an atypical subtrochanteric femur fracture healing without surgical intervention and only the addition of teriparatide treatment [51]. Conversely, a more contemporary study evaluating 24 months of teriparatide treatment in patients with atypical femur fractures showed no difference in fracture healing, hip bone mineral density, or trabecular bone score, but there was active bone formation on histomorphometry [52]. More research is needed for clear recommendations of teriparatide's use in AFFs, both acutely and in the setting of delayed union, but what is clear is that all patients should stop bisphosphonate use and continue calcium and Vitamin D supplementation [42, 48]. Due to their inherent risk of delayed union secondary to alterations in normal bone healing physiology, treating atypical subtrochanteric femur fractures with cephalomedullary nailing, anatomic or slight valgus alignment, and maximizing the metabolic and mechanical environments will increase a patient's likelihood of fracture union.

## 6. Summary

Elderly hip fractures are projected to continue to increase in the coming decades, and, with increased fracture treatment, there inherently is the possibility for complications related to fracture healing, including implant failure and nonunion. Recognizing femoral neck, peritrochanteric, and atypical subtrochanteric femur fracture nonunions and having the tools to treat such complications are necessary for improved patient outcomes. A thorough nonunion assessment should always be performed if intervention is desired, consisting of radiographic, infectious, and metabolic evaluation. When an abnormality is discovered, treating this, whether it is culture specific antibiotics and hardware removal with antibiotic cement implantation in the setting of infection or correction of vitamin D, electrolyte, hormone, or other chemical imbalances or improvement in mechanical forces across the fracture, all must be appropriately addressed to increase rate of successful nonunion surgery. Arthroplasty continues to be a reliable treatment option in the setting of both femoral neck and peritrochanteric femur nonunions, as it gives elderly patients the ability to immediately weight bear and removes the physiologic burden of fracture healing. Ultimately, enough emphasis cannot be placed on the importance of an appropriate, acceptable index surgery, along with maximizing patient bone healing capabilities with nutritional supplementation, vitamin D, and calcium to improve the chances of fracture healing and decreasing risk factors of nonunion. Atypical subtrochanteric femur fractures are at increased risk of delayed healing and nonunion; therefore maximizing the mechanical and physiologic environment for fracture healing is paramount. More research is warranted prior to making strong recommendations regarding the use of teriparatide for nonunions.

## Figures and Tables

**Table 1:** Recommendation for initial metabolic laboratory evaluation in the setting of fracture nonunion, with associated common abnormalities and underlying disease processes. We recommend CBC, CMP, 25-hydroxyvitamin D, iPTH, and TSH, as well as CRP and ESR to rule out infection. Additional tests may be required and obtained by a treating endocrinologist depending on the initial laboratory screening results.

**Laboratory Test**	**Important Values**	**Common Nonunion Abnormalities**	**Associated Diseases or Causes for Abnormal Values**
**Complete blood count (CBC)**	White blood cell (WBC)	Elevated	Infection
****		Decreased	Immunosufficiency

**Sedimentation rate**	(ESR)	Elevated	Infection

**C-reactive protein**	(CRP)	Elevated	Infection

**Comprehensive metabolic panel (CMP)**	Calcium (Ca)	Decreased	Hypoparathyroidism
****			Vitamin D deficiency
****			Renal osteodystrophy
****	Alkaline phosphatase (ALP)	Elevated	Vitamin D deficiency
****			Calcium deficiency
****			Chronic renal failure
****	Albumin (Alb)	Decreased	Protein deficiency
****	Glucose (Glu)	Elevated	Diabetes mellitus

**Thyroid function panel **	Thyroid stimulating hormone (TSH)	Elevated	Hypothyroidism (Low T3, T4)
****		Decreased	Hyperthyroidism (High T3, T4)
****	Free T4	Decreased	Hypothyroidism (Low T3, T4)
****		Elevated	Hyperthyroidism (High T3, T4)
****	Free T3	Decreased	Hypothyroidism (Low T3, T4)
****		Elevated	Hyperthyroidism (High T3, T4)

**Intact parathyroid hormone (iPTH)**	(iPTH)	Elevated	Primary hyperparathyroidism: pituitary adenoma
****			Secondary hyperparathyroidism: Vitamin D deficiency, Vitamin D resistance
****			Renal osteodystrophy

**25-hydroxyvitamin D**	(Vit D 25(OH))	Decreased	Vitamin D deficiency
****			Malabsorption/Liver disease
****			Anticonvulsant medication
